# Metabolic profiling analysis of the vitamin B_12_ producer *Propionibacterium freudenreichii*

**DOI:** 10.1002/mbo3.1199

**Published:** 2021-05-25

**Authors:** Jiao Liu, Yongfei Liu, Jie Wu, Huan Fang, Zhaoxia Jin, Dawei Zhang

**Affiliations:** ^1^ Tianjin Institute of Industrial Biotechnology Chinese Academy of Sciences Tianjin China; ^2^ College of Biotechnology Tianjin University of Science & Technology Tianjin China; ^3^ Key Laboratory of Systems Microbial Biotechnology Chinese Academy of Sciences Tianjin China; ^4^ School of Biological Engineering Dalian Polytechnic University Dalian China; ^5^ University of Chinese Academy of Sciences Beijing China

**Keywords:** medium optimization, metabolomics, *Propionibacterium freudenreichii*, vitamin B_12_ production

## Abstract

Vitamin B_12_ (VB_12_) is an indispensable cofactor of metabolic enzymes and has been widely used in the food and pharmaceutical industries. In this study, the effects of medium composition on VB_12_ production by *Propionibacterium freudenreichii* were evaluated and optimized based on statistical experiments. The results showed that glucose, yeast extract, KH_2_PO_4_, and glycine have significant effects on VB_12_ production. The final titer of VB_12_ reached 8.32 ± 0.02 mg/L, representing a 120% increase over the non‐optimized culture medium. We employed a metabolomics approach to analyze the differences of metabolite concentrations in *P*. *freudenreichii* cells cultivated in the original medium and optimized fermentation medium. Using multivariate data analysis, we identified a range of correlated metabolites, illustrating how metabolomics can be used to explain VB_12_ production changes by corresponding differences in the overall cellular metabolism. The concentrations of many metabolic intermediates of glycolysis, the Wood–Werkman cycle, the TCA cycle, and amino acid metabolism were increased, which contributed to the synthesis of propionic acid and VB_12_ due to an improved supply of energy and precursors.

## Graphical abstract

The metabolomics approach was employed to analyze the differences of metabolite concentrations in *Propionibacterium freudenreichii* cultivated in different media, explaining the differences in growth, fermentation characteristics, and vitamin B_12_ (VB_12_) production. Using multivariate data analysis, we illustrated how metabolomics can be used to explain VB_12_ production changes by corresponding differences in the overall cellular metabolism. The key metabolic nodes of VB_12_ synthesis in *P. freudenreichii* were identified, which may contribute to the metabolic engineering of this organism to improve industrial VB_12_ production.

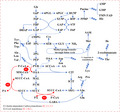

## 1 INTRODUCTION

Vitamin B_12_ (VB_12_, cobalamin), the only vitamin containing a metal element, is an essential cofactor of key enzymes that catalyze crucial biological activities such as the synthesis of DNA, amino and fatty acids in living organisms (And & Ragsdale, 2003; Eschenmoser, 1988; Fang et al., 2017). However, humans and other animals cannot synthesize VB_12_ de novo (Nielsen et al., 2012). Accordingly, nutritional deficiencies of VB_12_ can lead to a variety of complications in humans, including neuropsychiatric symptoms and various forms of cancer (Berg et al., 2013; Lechner et al., 2005). Therefore, VB_12_ has been widely used in the pharmaceutical, food, and feed industries, due to its special nutritional and economic value.

**FIGURE A1:**
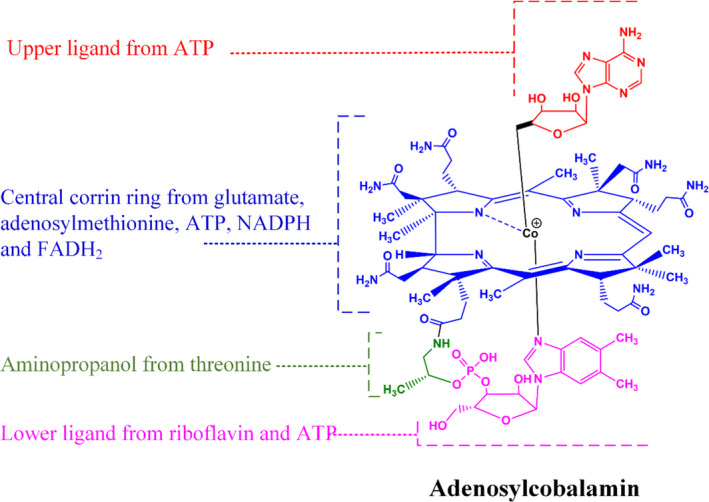
Molecular structure of VB_12_

The industrial production of VB_12_ is mainly dependent on the fermentation of microorganisms that can produce VB_12_ via a de novo synthesis pathway (Kojima et al., 1993; Rodionov et al., 2003; Swithers et al., 2012). *Pseudomonas denitrificans* and *Propionibacterium freudenreichii* are the most widely used VB_12_ producers in industrial applications (Martens et al., 2002). Previous reports revealed two very complex biosynthetic pathways of VB_12_, named the aerobic and anaerobic pathway, both of which comprise at least 25 steps with uroporphyrinogen III as the same initial precursor (Warren et al., 2002). The adenosylcobalamin molecule consists of three parts: the upper ligand, the central corrin nucleus, and the lower ligand (Figure A1). Fang et al. (2017) reviewed both pathways of VB_12_ biosynthesis. The first committed precursor of the central corrin nucleus is 5‐aminolaevulinic acid, which can be synthesized either from glycine and succinyl‐CoA in the C4 pathway or from glutamate in the C5 pathway. Uroporphyrinogen III is synthesized from eight molecules of 5‐aminolaevulinic acid. After cobalt chelation and eight peripheral methylation reactions with adenosylmethionine as the methyl donor, cob(II)yrinic acid a,c‐diamide is formed. Then, the upper ligand is attached to the cobalt atom of cob(II)yrinic acid a,c‐diamide to form adenosyl cobyrinic acid a,c‐diamide. After four stepwise amidation reactions at different carboxyl groups, adenosyl cobyric acid is produced from adenosyl cobyrinic acid a,c‐diamide. The lower ligand of VB_12_ is derived from aminopropanol, nicotinic acid mononucleotide, and DMBI. Although *P*. *freudenreichii* can *de novo* synthesize DMBI from riboflavin, oxygen is required for this process (Fang et al., 2017). Thus, *Propionibacterium* needs both anaerobic and aerobic conditions for effective vitamin B_12_ production. Furthermore, many studies added DMBI to the medium as an important precursor (Guo & Chen, 2018).

Adenosylcobalamin is coproduced by *P*. *freudenreichii* together with the main product propionic acid (PA). Many studies attempted to increase the production of PA and VB_12_ in *Propionibacterium* (Belgrano et al., 2018; Suwannakham & Yang, 2005). Several studies had attempted to increase VB_12_ production by adding some precursors such as cobalt ions (Quesada‐Chanto et al., 1994; Seidametova et al., 2004; Yongsmith et al., 1982) and DMBI (Marwaha & Sethi, 1984). In addition to these two factors, other media components also a play significant role in VB_12_ production, including the carbon source, yeast extract, casein hydrolysate, calcium pantothenate, NaH_2_PO_4_, and so on. Accordingly, many research efforts on improving the synthesis of VB_12_ are based on optimizing fermentation processes to reduce cell growth inhibition by PA, such as cell immobilization and in situ product removal. (Peng et al., 2012; Peng et al., 2012). The anaerobic synthesis pathway of VB_12_ in *Propionibacterium* has been studied intensively. Several reports showed that VB_12_ production could be increased by the homologous or heterologous overexpression of genes located in the *hem*, *cob*, and *cbi* operons, or gene clusters involved in VB_12_ synthesis, but the concentration was limited to between 0.96 and 1.68 mg/L (Piao et al., 2004). Little progress has been made on improving the VB_12_ yield of *Propionibacterium*. Further studies are needed to discover the potential metabolic regulation mechanisms, which would help remove the bottlenecks of VB_12_ production in *Propionibacterium*.

As a powerful analytical tool, metabolomics has been used to investigate the global metabolism of *Propionibacterium* for improving PA production (Cardoso et al., 2007; Choi & Mathews, 1994). Guan et al. found the key metabolic nodes affecting PA production by comparing metabolic profiles of wild‐type and genome‐shuffled mutant strains of *Acidipropionibacterium acidipropionici*, and then, the addition of key exogenous metabolites (precursors and amino acids) could increase the PA titer from 23.1 ± 1.2 to 35.8 ± 1.0 g/L (Guan et al., 2015). However, metabolomic studies of *P*. *freudenreichii* for enhancing VB_12_ production have not been reported.

In this study, *P*. *freudenreichii* CICC 10019 was used for VB_12_ production with the fermentation medium reported by Peng et al. (2012) as the original medium. As reported before, glycerol and amino acids, such as glycine, may be potential bottlenecks in VB_12_ biosynthesis. Thus, glycerol was added as the carbon source, yeast extract as the amino acid source, and glycine as the precursor of methyl group donors to optimize the fermentation medium. Fermentation medium optimization was first performed using a statistical experimental design to analyze the effects of key medium components on VB_12_ production by *P*. *freudenreichii*. Then, comparative metabolic profiling was applied to investigate the metabolic changes of *P*. *freudenreichii* grown on the original and the optimized fermentation medium. We then analyzed the intracellular metabolite concentrations at three time points of the fermentation process (logarithmic growth phase, stationary phase, and VB_12_ production phase) in the two media. This study deepens our understanding of the metabolic regulation of VB_12_ synthesis in *P*. *freudenreichii*.

## 2 MATERIALS AND METHODS

### 2.1 Microorganism, medium, and culture conditions

*Propionibacterium freudenreichii* CICC10019 was purchased from the Chinese Industrial‐Microorganism Conservation Center and stored in a pre‐culture medium supplemented with 20% glycerol at −80°C.

Agar‐based solid medium contained 20 g/L glucose, 10 g/L corn steep liquor, 2 g/L KH_2_PO_4_, 2 g/L (NH_4_)_2_SO_4_, 0.005 g/L cobalt chloride, and 20 g/L agar. The pre‐culture medium contained 35 g/L glucose, 21 g/L corn steep liquor, 4 g/L KH_2_PO_4_, 5 g/L (NH_4_)_2_SO_4_, and 0.005 g/L cobalt chloride. (Peng, Wang, Liu, et al., 2012) The original fermentation medium contained 60 g/L glucose, 40 g/L corn steep liquor, 4.6 g/L KH_2_PO_4_, and 0.0127 g/L cobalt chloride. (Peng, Wang, Liu, et al., 2012) The pH of the media was adjusted to 6.8–7.0 by adding 4 M NaOH.

Fermentations were performed in an anaerobic box (MGC C‐31; Mitsubishi Gas Chemical Co., Inc., Tokyo, Japan). An aliquot of the cryopreserved cells of *P*. *freudenreichii* CICC10019 was streaked on an agar‐based solid medium and incubated at 30°C for 5 days to obtain single clones of the activated strain. Then, the single clones were used to inoculate 100‐mL sealed anaerobic bottles containing 90 mL pre‐culture medium and grown for 24 h at 30°C. Finally, fermentation in 100 mL flasks containing 90 mL fermentation medium was inoculated with 10% (v/v) of the pre‐cultures and grown at 30°C for 122 h. The pH value should be adjusted to approximately 7.0 with NaOH solution every 12 h until the end of the fermentation. After 84 h, 5,6‐dimethylbenzimidazole was added to a final concentration of 0.9 mg/L, and the fermentations were finished at 122 h.

### 2.2 Medium optimization

Medium optimization was performed in two steps using Design Expert 10.0 software (Stat‐Ease, Inc., MN, USA). Firstly, a Plackett–Burman design was first used to determine the effects of eight variables on VB_12_ production. Eleven variables, including 8 medium components (A, glucose; B, corn steep liquor; C, glycerol; E, yeast extract; F, 1% CoCl_2_·6H_2_O; G, KH_2_PO_4_; I, (NH_4_)_2_SO_4_; J, glycine) and three dummy variables (D, H, K), were screened in twelve trials. The experiments were carried out according to the matrix obtained using Design Expert 10.0 as shown in Table A1. Next, four independent variables that showed significant correlations (glucose, yeast extract, KH_2_PO_4_, glycine) were optimized using a Box–Behnken design. The experimental design, model calculation, graph drawing, and other analyses were performed using Design Expert 10.0. The design matrix and responses are shown in Table A3. A total of 29 experiments were carried out to accurately estimate the errors in the response surface methodology model, and the center point in the design was repeated five times. Each obtained response was used to develop the model of the response. A quadratic polynomial model was applied to evaluate the response of the dependent variables using the following equation:

(1) $$Y_{i}=\beta_{0}+\sum_{i=1}^{4}\beta_{i}X_{i}+\sum_{i=1}^{4}\beta_{\mathrm{ii}}X_{i}^{2}+\sum_{i=1}^{3}\sum_{i=1}^{4}\beta_{\mathrm{ij}}X_{i}X_{j}$$

where *Y_i_* is the response value, *X_i_* is the coded value of the factor, *β*_0_ is a constant coefficient, *β_i_* is the linear coefficient, *β_ii_* is the quadratic coefficient, and *β_ij_* is the interaction coefficient (Box et al., 1978; Rodrigues et al., 2012). *X*_1_, *X*_2_, *X*_3_, and *X*_4_ represent the concentrations of glucose, yeast extract, KH_2_PO_4_, and glycine, respectively.

The final optimized fermentation medium contained 54.3 g/L glucose, 30 g/L corn steep liquor, 17.6 g/L yeast extract, 2.7 g/L KH_2_PO_4_, 3.5 g/L glycine, 0.005 g/L CoCl_2_·6H_2_O, 2 g/L (NH_4_)_2_SO_4_. The pH value of all media was adjusted to a value between 6.8 and 7.0 by adding 12% NaOH solution before autoclaving.

### 2.3 Metabolomic samples, quenching, and metabolite extraction

The samples for metabolic profiling analysis with the same amount of biomass were collected at the logarithmic phase (31.5 h), stationary phase (56 h), and VB_12_ production phase (106 h). The cellular metabolism was quenched by immediately adding the samples into 1 mL of 40% methanol (−20°C) and mixing gently. Then, the cells were pelleted by centrifugation (4,000 *g*, 4°C, 1 min), resuspended in 0.8 mL methanol (−80°C), and rapidly frozen in liquid nitrogen. For analysis, the cells were harvested by centrifugation of the quenched mixture for 10 min at 0°C, and the supernatant was collected. Then, the cells were resuspended in 50% methanol (−40°C). The mixtures were frozen in liquid nitrogen and thawed three times. After centrifugation (10,000 *g*, 4°C, 10 min), the supernatant was collected. The pellet was resuspended in 50% acetonitrile (−40°C), sonicated for 10 min in an ice bath, and then centrifuged (10,000 *g*, 4°C, 10 min). Finally, all the supernatants were combined in a single tube, filtered through a Teflon filter with 0.22 μm pore‐size, lyophilized overnight, and stored at −80°C until LC‐MS/MS analysis.

### 2.4 LC‐MS/MS analysis

The lyophilized samples were resuspended in 100 μl 50% acetonitrile and mixed by vortexing. Five microliters of the resulting sample were injected into a Shimadzu Nexera 30A ultra‐performance liquid chromatography instrument (Shimadzu, Kyoto, Japan) coupled with a TripleTOF™ 5600 mass spectrometer (Applied Biosystem Sciex, USA). LC separation was performed on a SeQuant ZIC‐HILIC column (100 × 2.1 mm, 3.5 μm, Merck, Germany) using a gradient of 10 mM ammonium acetate (A) and 100% acetonitrile (B). The samples were eluted at a flow rate of 0.2 mL/min with a gradient encompassing 90% B for 3 min, 90%–60% B for 3 min, 60%–50% B for 19 min, 50% B for 5 min, 50%–90% B for 1.5 min, and 90% B for 7.5 min.

An electrospray ionization source was used for detection in the negative mode under the following conditions: ion voltage, 4500 V; declustering potential, 80 V; source temperature, 550°C; curtain gas pressure, 35 psi; nebulizer gas pressure, 55 psi; heater gas pressure, 55 psi; mass acquisition at m/z 30–1200 for TOF MS and mass acquisition at m/z 50–1200 for MS/MS. The scan period for each sample included one TOF MS survey scan and eight MS/MS scans. The mass accuracy was calibrated using the automated calibrant delivery system (AB Sciex, Concord, Canada) connected to the second inlet of the DuoSpray source. Metabolite identification was performed according to the protocol described by Li et al. (2016). Briefly, MS and MSMS data were exported to identify putative metabolites by searching their accurate masses against the E. coli Metabolome Database. Then, the accuracy of mass measurement, isotopic fit, and LC peak quality were evaluated. Metabolites were identified using the following criteria: precursor mass accuracy <15 ppm; sufficient isotopic fit and good LC peak shape; fragments in the MSMS spectra match those in the in‐house library or online databases.

### 2.5 Pathway enrichment analysis

MetaboAnalyst (https://www.MetaboAnalyst.ca/) was used for further metabolic pathway enrichment analysis of the differential metabolites to obtain insights into the metabolic regulation in response to medium optimization. In the MetaboAnalyst analysis, Over Representation Analysis was set to “Hypergeometric Test” and Pathway Topology Analysis was set to “Relative‐betweenness Centrality.” The closest available pathway library was Escherichia coli K‐12 MG1655. The significant pathways were also identified based on a *p*‐value <0.05 and FDR correction <0.05 and listed in descending order according to the value of the “Impact” factor, while those with an Impact value of zero were excluded.

### 2.6 Other analytical methods

The optical density (OD) was determined using a V‐1600 spectrophotometer at 600 nm. The total concentration of the whole VB_12_ both inside and outside of cells was determined by HPLC as previously described by Fang et al (Fang et al., 2018). The fermented broth (20 mL) was mixed with 2 mL of 8% (w/v) NaNO_2_ and 2 mL of glacial acetic acid, after which the mixture was boiled for 30 min and filtered through a Teflon filter with 0.22 μm pore‐size. The final sample was resolved on a reverse‐phase C‐18 column (4.6 × 250 mm, 5 µm, Agilent) using an Agilent 1260 HPLC operating at 30°C and monitored at 361 nm. The mobile phase consisted of water and methanol (70:30 [v/v]) at a flow rate of 0.8 mL/min. The VB_12_ standard was from Sigma‐Aldrich (USA).

Organic acids and glucose present in the fermentation broth samples were analyzed by HPLC equipped with an organic acid analysis column (HPX‐87H, Bio‐Rad) operated at 55°C with 5 mM H_2_SO_4_ as the mobile phase at 0.5 mL/min. Organic acids and glucose standards were used to create a calibration curve. Averages of three biological replicates were reported.

### 2.7 Statistical analysis

In this study, three independent experiments were carried out for each condition in medium optimization, while four biological replicates were used to perform metabolic analysis for each sample condition. The experimental data were reported as the mean value with the error indicated by the standard deviation. Metabolomics data were normalized and analyzed for statistical significance using MATLAB and Excel software (Microsoft Corp., USA). The normalized data were imported into SIMCA‐P software (Ver 14.1; Umetrics, Umea, Sweden) for multivariate statistical analysis. PCA was conducted after mean‐centering and unit variance scaling. Hierarchical clustering analysis was performed using MeV software.

## 3 RESULTS

### 3.1 Screening of significant variables for VB_12_ production using a Plackett–Burman design

To identify the factors that significantly affect VB_12_ biosynthesis, eight components of the fermentation medium were selected for the Plackett–Burman design, and the low and high levels of each factor were listed in Table A1. As shown in Table A2, factors with confidence levels above 95% (*p* < 0.05) were considered to have significant effects. The results showed that glucose (*p* = 0.0021), yeast extract (*p* = 0.0007), KH_2_PO_4_ (*p* = 0.0102), and glycine (*p* = 0.0256) all have significant effects on VB_12_ production. Table A2 shows the regression coefficient for each factor. A positive regression coefficient indicates that a higher level of the factor has a beneficial effect on the production of VB_12_, while a negative regression coefficient indicates the opposite relationship. High levels of glucose (40 g/L), yeast extract (10 g/L), and glycine (2 g/L) had an apparent positive effect on VB_12_ production, while a low level of KH_2_PO_4_ (3 g/L) was also beneficial for VB_12_ production. Then, these four components were selected for further experiments. The concentrations of the other factors in the medium for further optimization were set as follows: corn steep liquor 30 g/L, CoCl_2_·6H_2_O 0.005 g/L, and (NH_4_)_2_SO_4_ 2 g/L.

### 3.2 Model fitting and statistical analyses

To obtain a better medium formulation for VB_12_ production, we used response surface methodology to further optimize the concentrations of the above four factors. A Box–Behnken design with four independent variables was chosen, and each variable was assessed at three levels: −1 (the concentration preceding the optimal value in the previous experiment), +1 (the concentration following the optimal value in the previous experiment), and 0 (the average of the −1 and +1 concentrations). Table A3 lists all 29 experimental runs of the Box–Behnken design and the corresponding results. The maximal (8.3 mg/L) and minimal (4.6 mg/L) concentrations of VB_12_ were obtained in runs No. 28 and 2, respectively. The maximal concentration of VB_12_ obtained in run No. 28 was higher than the (3.8 mg/L) obtained in the original medium, which confirmed the positive effects of the tested components on VB_12_ production.

**FIGURE 1:**
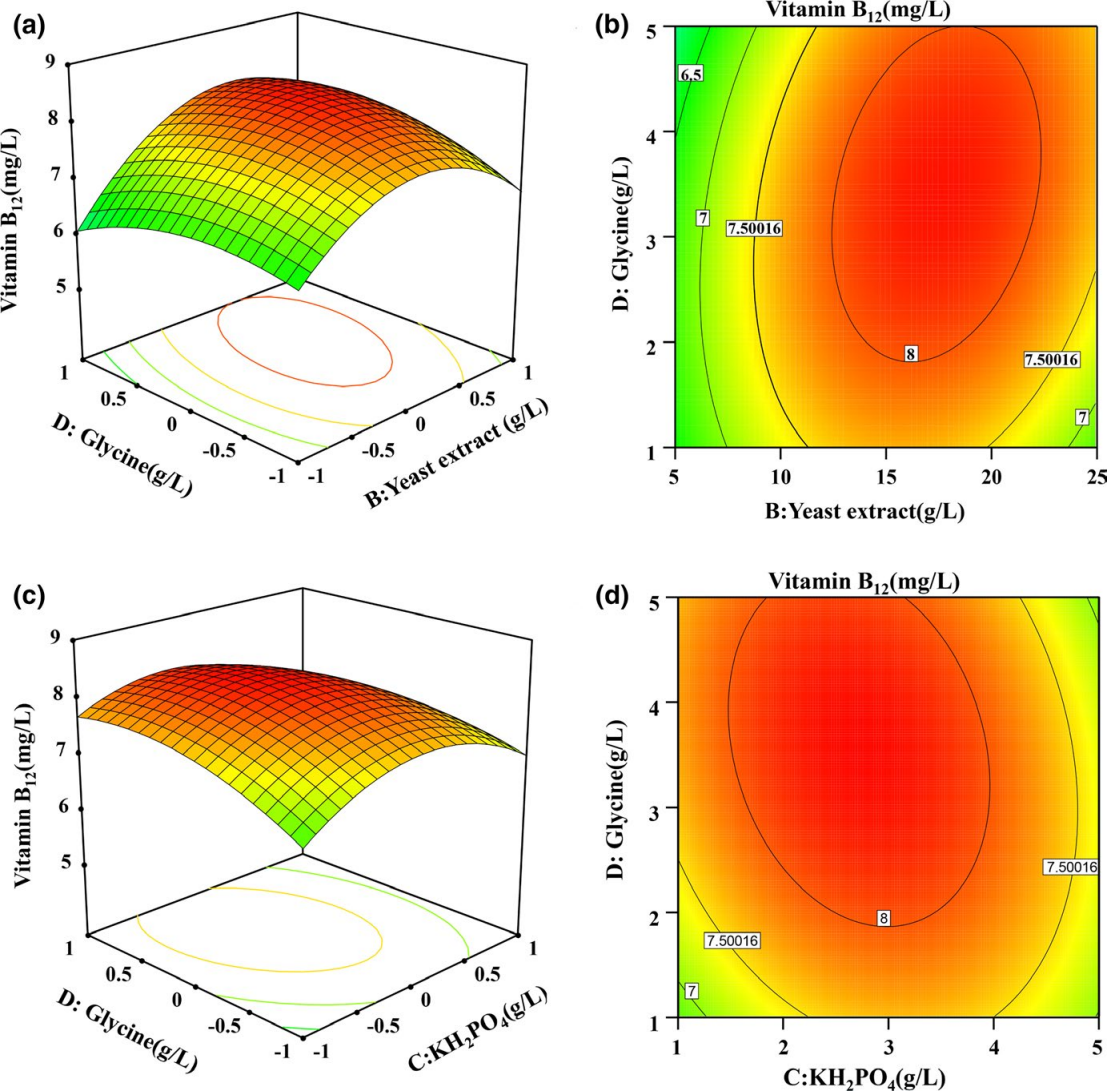
Response surface for the production of VB_12_ by *P*. *freudenreichii* grown at 30°C for 122 h, with pH adjustment to 7.0 every 12 h, according to the Box–Behnken design. Response surface (a) and contour plot (b) of the combined effects of yeast extract and glycine on VB_12_ production. Response surface (c) and contour plot (d) of the combined effects of KH_2_PO_4_ and glycine on VB_12_ production

The quadratic regression equation was applied for further data analyses, and the Lack‐of‐Fit test (Lack of Fit *F* value = 0.1183) and *R* ^2^ summary statistics (adjusted *R* ^2^ = 0.9728, predicted *R* ^2^ = 0.9275) indicated a good fit. The significance of the response surface methodology model was evaluated using analysis of variance (ANOVA), as shown in Table A4. It was found that all linear and quadratic effects of glucose, yeast extract, KH_2_PO_4_, and glycine should be significant in the model (*p* < 0.05). The results also indicated that there are significant interactions between glycine and yeast extract as well as between glycine and KH_2_PO_4_. Then, the three‐dimensional response surface graphs were plotted to illustrate the optimum levels of the tested components for each pair of factors by keeping the other two factors constant at their middle level (Figure 1). When the concentration of yeast extract was increased from 5 to 25 g/L, VB_12_ production first increased and then decreased (Figure 1a). KH_2_PO_4_, like yeast extract, showed a strong effect on VB_12_ production. By contrast, the concentration of glycine had little effect on VB_12_ production (Figure 1c), which was consistent with the ANOVA analysis (Table A4). A simplification of the model was further performed by removing the items that were not significant at the 95% confidence level in the quadratic model equation. Then, a new ANOVA for the simplified model was conducted, and the results are shown in Table A5. The yeast extract concentration had the strongest effect, followed by glucose, KH_2_PO_4_, and glycine concentrations. The final simplified model was significant (*F* = 106.40, *p* < 0.01) and had a good fit (the adjusted *R* ^2^ was 0.9834, and the predicted *R* ^2^ was 0.9741). The model for VB_12_ production was represented using Equation (2):

(2) $$\left[{\text{VB}}_{12}\right]=8.2‐0.17A+0.44B‐0.15C+0.11D+0.32\text{BD}‐0.24\text{CD}‐1.97A^{2}‐1.05B^{2}‐0.76C^{2}‐0.4D^{2}$$

The maximum point of Equation (2) should be in a downward open parabola due to negative signs of all the quadratic coefficients. Thus, the best concentrations of all the factors in Equation (2) were calculated as follows: glucose (A) 54.3 g/L, yeast extract (B) 17.6 g/L, KH_2_PO_4_ (C) 2.7 g/L, and glycine (D) 3.5 g/L. The model predicted a maximum of 8.283 mg/L VB_12_.

### 3.3 Comparative analysis of VB_12_ fermentation using the original and optimized media

**FIGURE 2:**
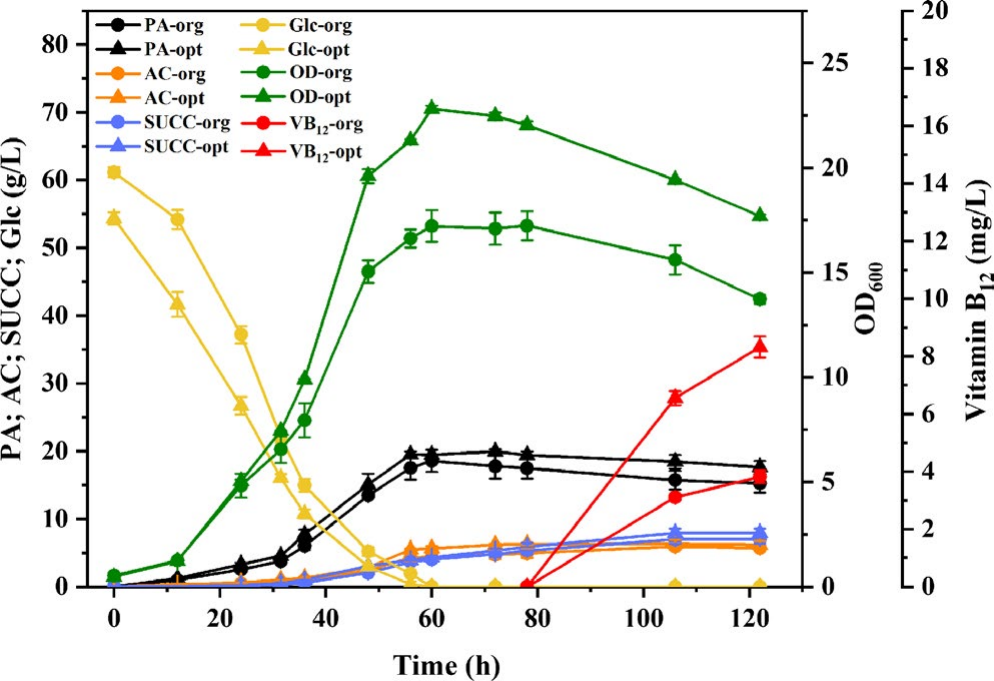
VB_12_ fermentation in anaerobic flasks using the original and optimized medium at 30°C for 122 h, with pH adjustment to 7.0 every 12 h. Green: biomass, yellow: glucose concentration, black: propionic acid concentration, blue: succinic acid concentration, orange: acetic acid concentration, and red: VB_12_ concentration. Solid circles represent the original medium (org), and triangles represent the optimized medium (opt). Data were presented as means * standard deviations (SD) calculated from three independent experiments

To verify the validity of Equation (2), fermentation of *P*. *freudenreichii* was performed using the predicted fermentation conditions (repeated 3 times). The final titer of VB_12_ reached 8.32 mg/L (0.47 mg/L/OD) (Figure 2) and was inside the 95% confidence interval (8.03–8.36 mg/L) of the predicted value of Equation (2). Thus, it could be concluded that the simplified model was valid. The whole fermentation process could be divided into three phases: phase I (0–48 h; logarithmic growth phase), phase II (48–78 h; stationary phase), and phase III (78–122 h; VB_12_ production phase). In phase I, biomass increased quickly with the consumption of large amounts of nutrients, and then, the cell growth slowed down with concomitant generation of large amounts of organic acids in phase II, the central corrin nucleus of VB_12_ was predominantly produced in phases I and II, while biomass decreased in phase III, and the VB_12_ synthesis started when DMBI was added. When the fermentation was finished, the final titer of VB_12_ in the optimized group reached 8.32 ± 0.02 mg/L, which was 2.2‐fold higher than in the original group (3.81 mg/L). The titer of VB_12_ per unit OD also increased from 0.28 mg/L/OD (original group) to 0.47 mg/L/OD (optimized group). Furthermore, the concentrations of propionic, succinic, and acetic acid in the optimized group were 17.6, 7.9, and 6.8 g/L, which was slightly higher than the corresponding values of the original group, which were 15.3, 7.0, and 5.7 g/L, respectively.

### 3.4 Comparative metabolic profiling analysis of VB_12_ production in the original and optimized groups

**FIGURE 3:**
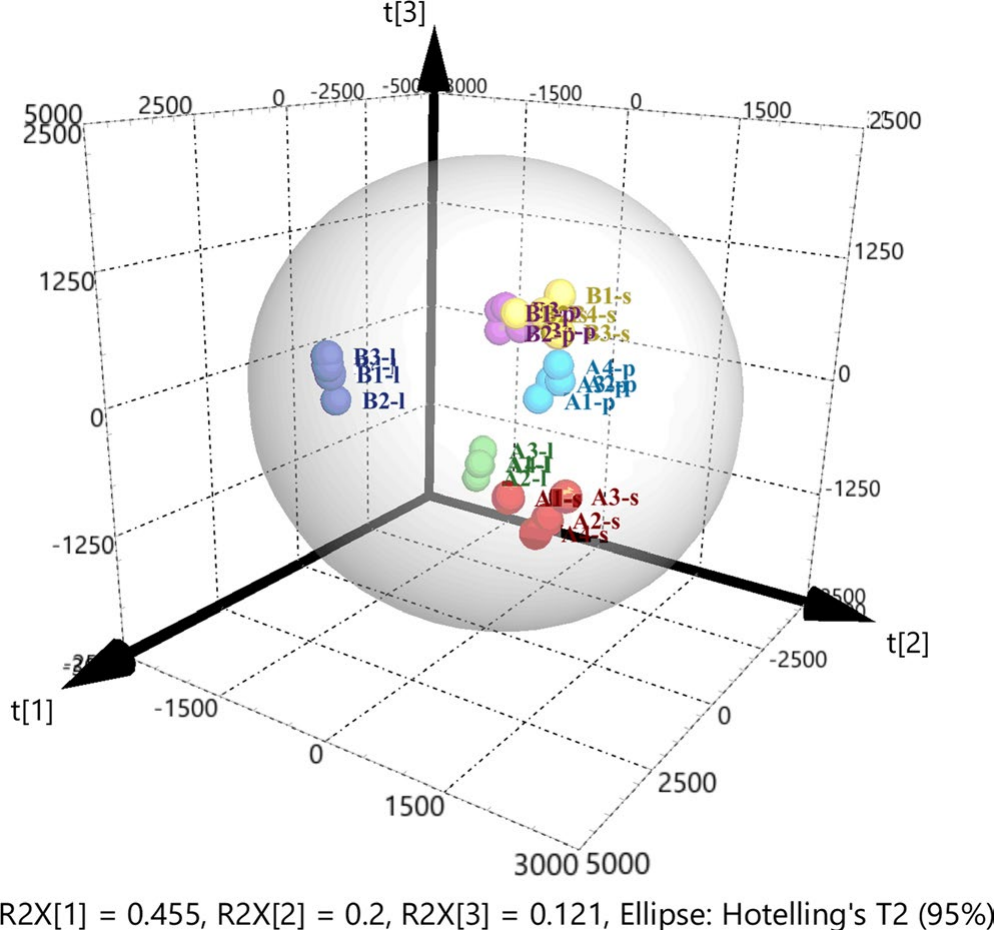
PCA‐score scatter 3D plot of metabolic profile of *P*. *freudenreichii* grown in an original and optimized medium at 30°C for 122 h, with pH adjustment to 7.0 every 12 h. “A” and “B,” respectively, indicated the fermentation samples in the original and optimized medium. “l,” “s,” and “p,” respectively, mean the fermentation samples obtained at the logarithmic phase (31.5 h), stationary phase (56 h), and VB_12_ production phase (106 h)

Metabolic profiles of *P*. *freudenreichii* were analyzed at 31.5, 65, and 106 h of fermentation in original and optimized media to explain how metabolic regulation of the cell affects VB_12_ synthesis. As shown in Table A6, a total of 69 intracellular metabolites including sugars, organic acids, amino acids, and other compounds were detected and then identified via LC‐MS/MS. Then, principal component analysis (PCA) was first applied to explore the metabolic data as shown in Figure 3. The *R* ^2^ and *Q* ^2^ of the PCA were 0.777 and 0.502, respectively, indicating that the model had reached a good fitting degree. As shown in the score plot of the PCA, four parallel samples from the same time point of VB_12_ fermentation in the optimized or original medium were tightly clustered, and distinct separation was visible between the samples from different time points or different media. Thus, the metabolic data were suitable for further analysis of intracellular metabolism changes during VB_12_ fermentation in the optimized and original medium.

### 3.5 Different metabolites and pathway enrichment analysis

**FIGURE 4:**
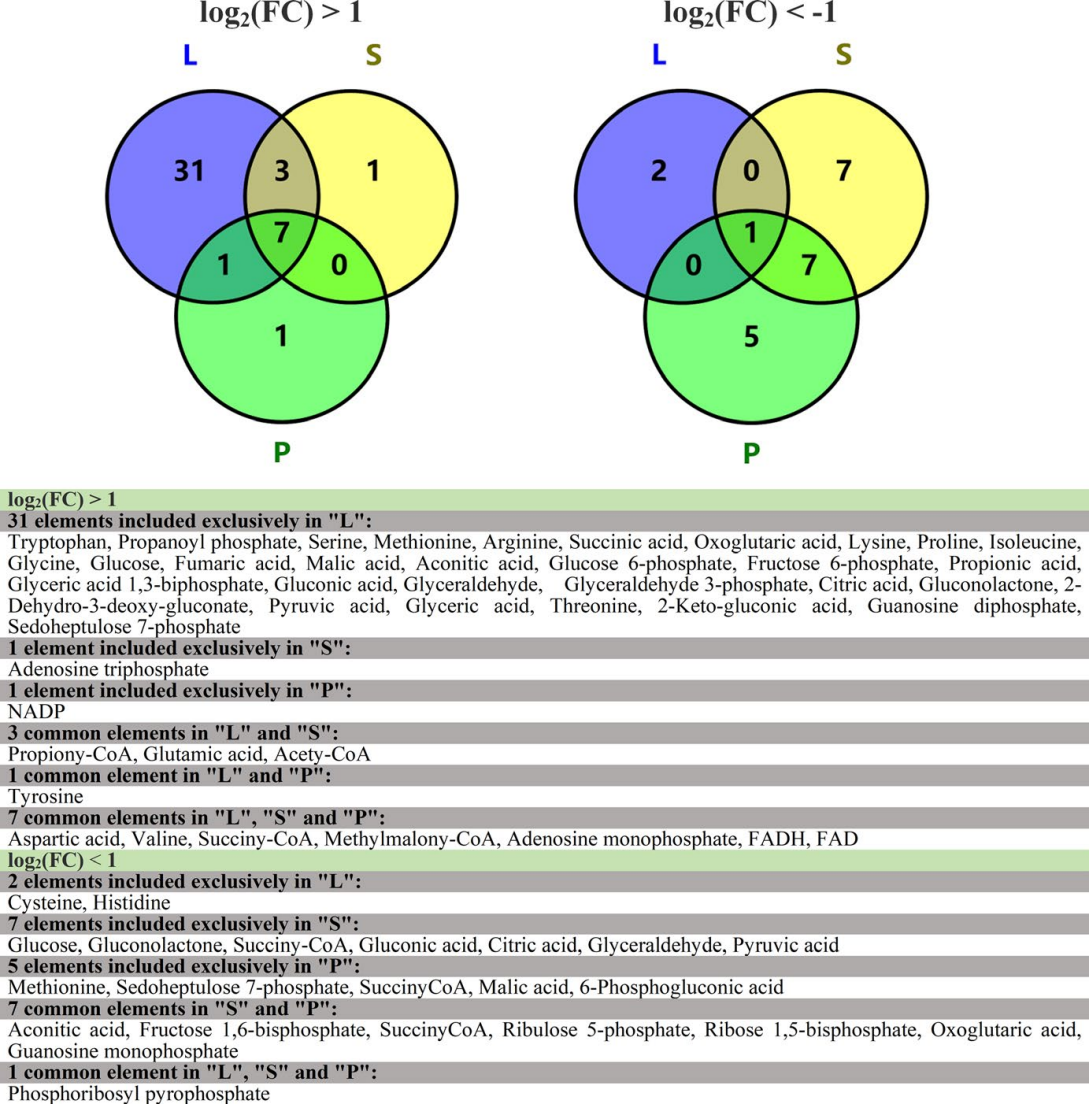
Venn diagrams of the upregulated (log_2_ (FC) > 1) and downregulated (log_2_ (FC) < −1) differential metabolites at the logarithmic phase (l), stationary phase (s) and VB_12_ production phase (p). log_2_ (FC) means the log base twofold change values. Differential metabolites were identified using the following criteria: log_2_ (FC) > 1 or <−1, and −log(P) > 1.301 (*p*‐value < 0.05)

The log base twofold change values (log_2_ (FC)) between the optimized and original groups were calculated for each time point. As shown in Table A7, differential metabolites were identified using the following criteria: log_2_ (FC) > 1 or <−1, and ‐log(P) > 1.301 (*p*‐value < 0.05). During the logarithmic growth phase, a total of 46 metabolites exhibited differential levels, 43 and 3 of which were, respectively, up‐ and downregulated in the optimized group. Similarly, 11 and 15 different metabolites were, respectively, up‐ and downregulated in the optimized group during the stationary phase, as well as 9 and 14 differential metabolites during the VB_12_ production phase. As shown in Figure 4, many more marker metabolites were upregulated, especially in the logarithmic growth and stationary phases, while relatively few were downregulated, mainly in the stationary and VB_12_ production phases. Next, MetaboAnalyst was used for further metabolic pathway enrichment analysis of the differential metabolites to obtain insights into the metabolic regulation in response to medium optimization.

**FIGURE 5:**
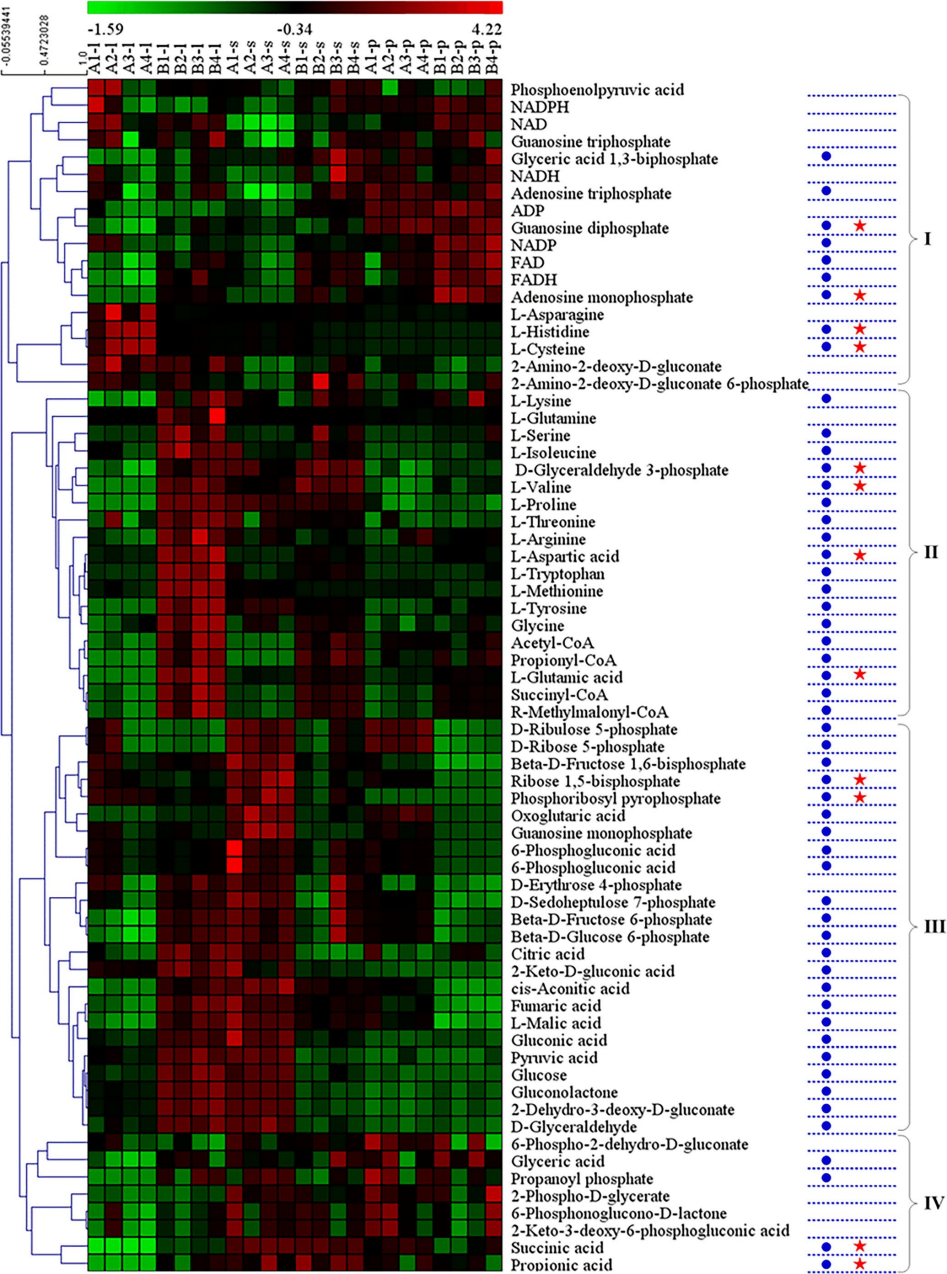
Hierarchical cluster analysis of all detected metabolites in the metabolic profile of *P*. *freudenreichii* grown in the original and optimized medium at 30°C for 122 h, with pH adjustment to 7.0 every 12 h. “A” and “B,” respectively, indicated the fermentation samples in the original and optimized medium. “l,” “s,” and “p,” respectively, mean the fermentation samples obtained at the logarithmic phase (31.5 h), stationary phase (56 h), and VB_12_ production phase (106 h). Blue circles indicate differential metabolites, and red stars indicate metabolites with a significant positive correlation between the two media. Four groups (I, II, III, and IV) are clustered

**FIGURE 6:**
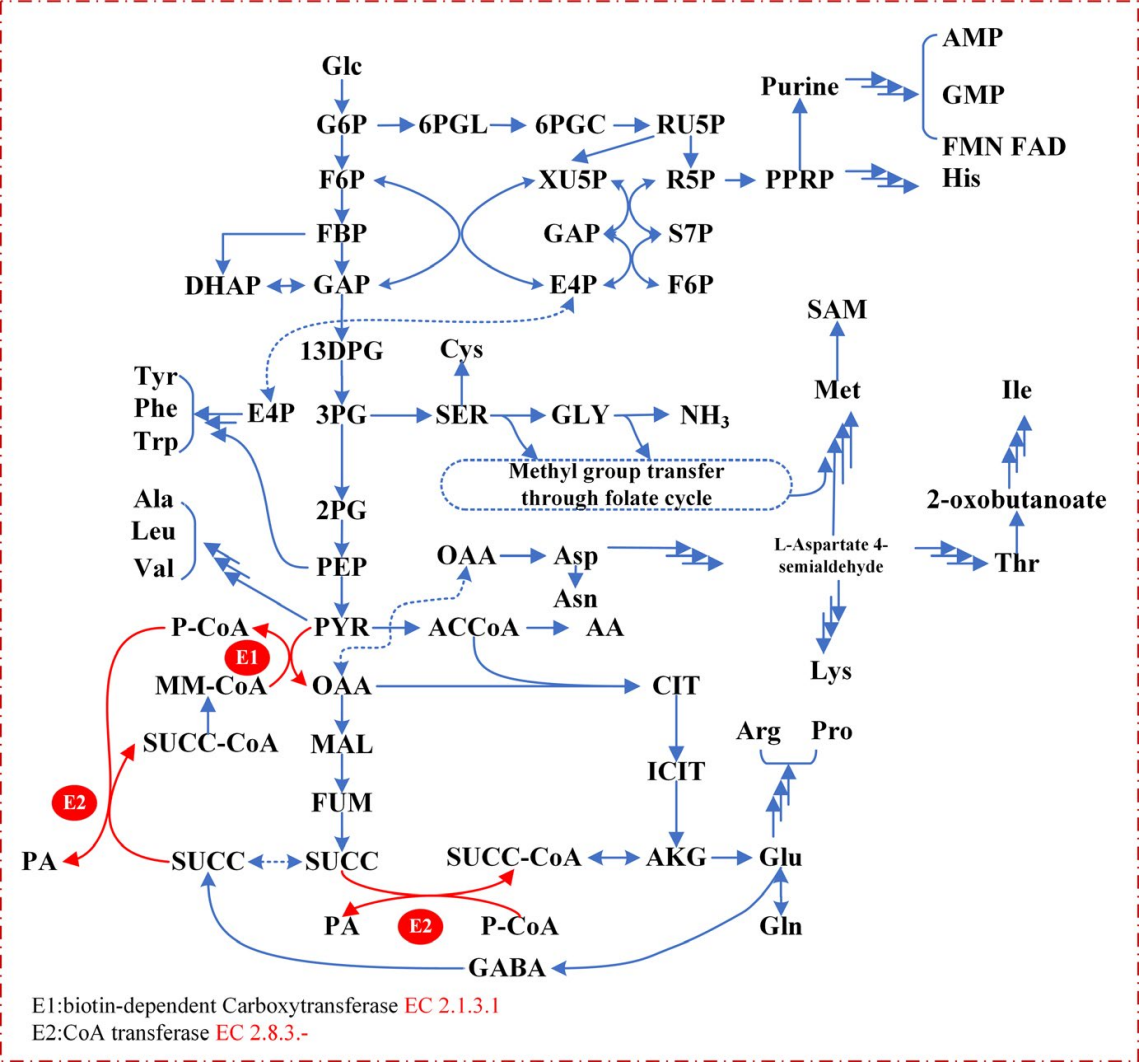
Metabolic pathways of PA biosynthesis in *P*. *freudenreichii*

During the logarithmic phase, almost all differential metabolites were upregulated in the optimized medium, while no significant pathway was enriched among the few downregulated metabolites. Thus, downregulation of metabolites failed to identify any metabolic pathways by enrichment analysis. The pathway analysis of upregulated metabolites is summarized in Table A8. The most significant metabolic pathways revealed by MetaboAnalyst were propanoate metabolism, glycine, serine and threonine metabolism, alanine, aspartate and glutamate metabolism, butanoate metabolism, citrate cycle, and glycolysis or gluconeogenesis. C1 metabolism (glycine and serine) and metabolic pathways that provide precursors for VB_12_ (glycine, serine, and threonine metabolism, alanine, aspartate, and glutamate metabolism) were more important revealed according to MetaboAnalyst. Therefore, the supplemental nitrogen source and glycine in the optimized medium promoted the PA metabolism and cell growth of *P*. *freudenreichii* and were also beneficial for the supply of precursors and methylation donors for the VB_12_ synthesis pathway. Secondly, the pathway analysis with MetaboAnalyst showed that medium optimization enabled the bacteria to maintain more active PA synthesis, which helped maintain higher cell activity and energy supply during the stationary phase (Tables A9‐A10). MetaboAnalyst indicated that the pentose phosphate pathway was downregulated. Finally, MetaboAnalyst obtained similar results for the up‐ and downregulated differential metabolites in the production phase and stationary phase (Table A11). The pentose phosphate pathway was identified as significant for downregulated differential metabolites. All the detected metabolites were subjected to hierarchical cluster analysis as shown in Figure 5. All metabolites were clustered into four categories (I, II, III, IV) according to their change trends in the different fermentation periods. Cluster I mainly included cofactors such as ATP and NADH; cluster II included various amino acids; cluster III mainly included the metabolites involved in glycolysis, pentose phosphate pathway, and the TCA cycle; and cluster IV was mainly related to PA synthesis. Only 12 metabolites exhibited positive correlations of trends between the optimized and original groups based on correlation coefficients, (red stars), which indicates that the trends of most differential metabolites were altered by medium optimization. As shown in the metabolic network of VB_12_ synthesis in *P*. *freudenreichii* (Figure 6), the synthesis of VB_12_ relies on various metabolic pathways, mainly providing cofactors (ATP, GTP, NADH, NAD, and FADH_2_) and amino acids (glutamic acid, glutamine, S‐adenosyl methionine, and threonine).

### 3.6 Analysis of key metabolic pathways associated with VB_12_ and PA production

Next, we analyzed the changes of glycolysis, pentose phosphate pathway, the TCA cycle, pyruvate metabolism, and different amino acid metabolism pathways according to the above results of pathway enrichment analysis, and analyzed their possible effects on VB_12_ and PA synthesis. The central metabolic pathways in *P*. *freudenreichii* were presented in Figure 6.

As shown in Figures 5 and 6, the metabolites involving glycolysis (glucose 6‐phosphate, fructose 6‐phosphate, fructose 1,6‐bisphosphate, glyceraldehyde 3‐phosphate, glycerate 1,3‐biphosphate, 2‐phospho‐d‐glycerate, phosphoenolpyruvate, and pyruvate) could be clustered into different groups according to their change trends during the fermentation time. Firstly, the levels of metabolites in the upstream part of the glycolysis (glucose 6‐phosphate, fructose 6‐phosphate, fructose 1,6‐bisphosphate) first rose and then dropped along during the entire fermentation in the original group, while they reached the peak at the very beginning and then decreased until the end of fermentation in the optimized group. Secondly, the downstream metabolites (glyceraldehyde 3‐phosphate, glycerate 1,3‐biphosphate, 2‐phospho‐d‐glycerate, phosphoenolpyruvate, and pyruvate) first rose and then dropped during the entire fermentation in both groups. The different behaviors of the up‐ and downstream parts of the glycolysis may be due to shared metabolites between the upstream part and the one with the pentose phosphate pathway. Normally, the pentose phosphate pathway provides precursors for purine and adenosine synthesis and plays an important role in bacterial ATP and DNA synthesis, so the addition of phosphate and other nutrients would decrease the flux through the pentose phosphate pathway and increase the levels of purine and adenosine, which was observed in the optimized group (Figure 5).

In *P*. *freudenreichii*, the TCA cycle and the Wood–Werkman cycle have many common intermediate metabolites, including acetyl‐CoA, succinic acid, fumaric acid, and malic acid. In addition, propionyl‐CoA, R‐methylmalonyl‐CoA, and PA exclusively take part in the Wood–Werkman cycle, while citric acid, cis‐aconitic acid, and oxoglutaric acid only take part in the TCA cycle. As can be seen in Figure 5, the intermediate metabolites of the Wood–Werkman cycle, especially the coenzyme intermediates, were significantly more abundant in the optimized medium than in the original medium, especially during the initial phase of fermentation. The production of PA by *P*. *freudenreichii* is characterized by growth coupling, and the energy generated during the formation of PA is beneficial to the growth of bacteria and the synthesis of VB_12_. The role of succinic acid in the PA synthesis pathway is consistent with the metabolic role of PA itself and is different from other intermediate metabolites, which may be attributed to the fact that succinic acid consumption and PA production are directly coordinated by the enzyme E2 (Figure 6). Therefore, the formation of succinic acid is the most important factor for PA synthesis. The metabolism of amino acids was significantly different in the two different media (Figure 5). Except for l‐asparagine, l‐cysteine, and l‐histidine, all other detected amino acids (l‐aspartic acid, l‐glutamine, l‐glutamic acid, l‐tryptophan, l‐serine, l‐threonine, l‐methionine, l‐valine, l‐isoleucine, l‐lysine, l‐arginine, l‐proline, l‐tyrosine, and l‐glycine) were significantly more abundant in the optimized group, which was due to the addition of yeast extract as a rapidly available nitrogen source for cell growth, which not only accelerated the growth of bacteria but also enhanced the conversion of the carbon source into precursors of VB_12_ synthesis. It is worth noting that in the early phase of fermentation, the bacterial cells in the original group contained more l‐asparagine, l‐cysteine, and l‐histidine than those in the optimized group. One of the most important physiological functions of l‐asparagine is to store and transport nitrogen, which may affect nitrogen metabolism and stress resistance. Cysteine can be produced from methionine, which is reported to be capable of storing reducing power and has a certain anti‐reverse effect. The metabolism of cysteine may also affect the formation of S‐adenosyl methionine (SAM) from methionine, which affects the synthesis of VB_12_. Histidine is synthesized from d‐ribulose 5‐phosphate in the pentose phosphate pathway, which is converted into phosphoribosyl pyrophosphate after several steps, in a pathway that is competitive with purine and adenosine. Higher histidine abundance in the original medium is likely due to higher flux through the pentose phosphate pathway. In particular, the VB_12_ synthesis process consumes large amounts of methyl groups, the direct source of which is SAM, while the synthesis of SAM depends on the methionine and folic acid cycle. The one‐carbon moiety that enters the folic acid cycle can be derived from glycine, histidine, serine, or cysteine. This is one of the main reasons why the addition of glycine and yeast extract in the optimized medium increased the yield of VB_12_. Finally, cofactors such as ATP and NAD(P)H also play important roles in the synthesis of VB_12_. In the optimized medium, especially during the period of VB_12_ production after the growth phase, the total intracellular cofactor content of the cells in the optimized medium was significantly higher than in the original medium. The increase in the overall abundance of cofactors is beneficial for the synthesis of VB_12_ and may also be related to the addition of phosphorus.

## 4 DISCUSSION

*Propionibacterium freudenreichii* has been used to produce therapeutically active VB_12_, and increasing the production of VB_12_ by *Propionibacterium* has received a lot of attention. Many studies optimized the fermentation medium by adding precursors of cobalamin biosynthesis to enhance VB_12_ production by *P*. *freudenreichii*, and these studies revealed DMBI and cobalt ions play crucial roles in the biosynthesis of VB_12_ (Murooka et al., 2005; Paulina et al., 2017; Roman et al., 2001; Wang et al., 2015; Thirupathaiah et al., 2012). Other media components were also optimized in many studies to improve the productivity of VB_12_ using statistical experimental designs. A 93% increase of VB_12_ concentration (4 mg/L) was obtained following medium optimization with glycerol as the carbon source (Kosmider et al., 2012). Five of 13 tested medium components (calcium pantothenate, NaH_2_PO_4_, casein hydrolysate, glycerol, and FeSO_4_) were found to have significant effects on VB_12_ production. Another 43% increase of VB_12_ production by medium optimization with glucose as the carbon source was also reported (Chiliveri et al., 2010). Optimization of the eight most significant influencing factors (FeSO_4_, inoculum size, (NH_4_) _2_HPO_4_, glucose, DMBI, yeast extract, sodium lactate, and CoCl_2_) was performed using the Taguchi method. Waste frying sunflower oil was used as a medium for VB_12_ production by *P*. *freudenreichii*, whereby DMBI, CoCl_2_, FeSO_4_, and calcium chloride were found to have the most significant effects on VB_12_ production during medium optimization (Hajfarajollah et al., 2014). As shown in Figure 6, other precursors such as glycine, threonine, 5‐aminolevulinic, or choline were also important for the production of VB_12_ (Piwowarek et al., 2018). Many compounds such as DMBI and cobalt ions are related to VB_12_ synthesis as direct precursors. Many studies have provided insights into the effects of these compounds on the synthesis of vitamin B_12_ with clear conclusions. Our study focused more on the effect of whole‐cell metabolic changes induced by precursor additives on VB_12_ synthesis. Thus, these compounds were not considered in our medium optimization. The original medium used for VB_12_ production was reported by Peng, Wang, Liu, et al. (2012), based on which glycerol, yeast extract, and glycine were added to the optimized medium in this study (Peng, Wang, Liu, et al., 2012).

Experimental results of the response surface design indicated that the yield of VB_12_ could be significantly increased by adequate supplementation of glucose, yeast extract, KH_2_PO_4,_ and glycine. Yeast extract contains a variety of amino acids, which are more easily available and fast‐acting nitrogen sources for *P*. *freudenreichii* than the corn steep liquor in the original medium. The additional KH_2_PO_4_ may promote the synthesis of metabolites containing phosphate moieties, such as metabolic cofactors and nucleic acids. Glycine acts as a primary methyl group donor for VB_12_ synthesis. According to the identified clusters and pathway analysis, the metabolism of *P*. *freudenreichii* underwent complex changes in response to the modified medium, mainly affecting the sugar metabolism pathways (glycolysis, pentose phosphate pathway, TCA cycle, and PA metabolism), amino acid metabolism, and cofactor metabolism. The changes in metabolic pathways not only affected the synthesis of VB12 but also led to changes in cell growth and PA production.

Usually, inhibiting PA biosynthesis or removing PA from the culture medium was necessary for improving VB_12_ production, but Wang et al. (2015) found that cobalamin production still requires a certain concentration of PA (10–20 g/L during the initial stage of fermentation and 20–30 g/L at a later stage) (Wang et al., 2015). It therefore seems that PA does not always have a negative effect on VB_12_ synthesis. Guan et al. (2015) investigated PA production in *P*. *freudenreichii* using a metabolomic approach, which revealed key metabolites and pathways that are positively correlated with PA production. Notably, these were surprisingly similar to the ones that promoted VB_12_ production in this study. The upregulation of glycolysis, pentose phosphate pathway, and the common metabolites between the TCA cycle and Wood–Werkman cycle benefited not only PA production as overflow metabolism but also VB_12_ synthesis due to increased supply of energy and precursors. It is worth noting that amino acid metabolism plays an important role in both PA and VB_12_ synthesis. Guan et al. (2015) found that PA production increased by 39.9% when 20 mM arginine and aspartate were added, while leucine, threonine, γ‐aminobutyric acid, citrulline, methionine, and serine had no significant effects on PA production (Guan et al., 2015). The strength of aspartate metabolism in *P*. *freudenreichii* was found to be highly strain‐dependent (Thierry et al., 2011). Aspartate can be deaminated to fumarate and further reduced to succinate, with concomitant production of NAD^+^ and ATP (Crow, 1986, 1987). The metabolism of other amino acids, such as glutamate, glycine, and threonine, can provide precursors for VB_12_ production. Overall, PA and VB_12_ are not produced by strictly competing pathways, but their synthesis is correlated in *P*. *freudenreichii*. For example, a high energy yield is provided by PA fermentation via the Wood–Werkman cycle for cell growth and VB_12_ synthesis (Thierry et al., 2011). As the cofactor for methylmalonyl CoA mutase, VB_12_ also plays an important role in the conversion of succinyl‐CoA into methylmalonyl‐CoA, which is a penultimate step of the Wood–Werkman cycle (Piwowarek et al., 2018).

## 5 CONCLUSIONS

A two‐step medium optimization method based on a statistical experimental design was applied to increase the VB_12_ production by more than 118%. High levels of glucose (40 g/L), yeast extract (10 g/L), and glycine (2 g/L) had an apparent positive effect on VB_12_ production, while a low level of KH_2_PO_4_ (3 g/L) was also beneficial for VB_12_ production. This study provides in‐depth insight into the central carbon metabolism of *P*. *freudenreichii*. The upregulation of glycolysis, the pentose phosphate pathway, amino acid metabolism, and the common metabolites between the TCA cycle and Wood–Werkman cycle benefited not only PA production as overflow metabolism but also VB_12_ synthesis due to increased supply of energy and precursors. PA metabolism indirectly contributes to VB_12_ synthesis through growth coupling, which depends on succinic acid.

## 6 ETHICS STATEMENT

None required.

## CONFLICT OF INTEREST

None declared.

## AUTHOR CONTRIBUTIONS

**Jiao Liu:** Conceptualization (equal); data curation (equal); writing–original draft (equal); writing–review and editing (equal). **Yongfei Liu:** Conceptualization (equal); data curation (equal). **Jie Wu:** Conceptualization (equal); data curation (equal); methodology (equal); writing–original draft (equal). **Huan Fang:** Methodology (equal); writing–review and editing (equal). **Zhaoxia Jin:** Conceptualization (equal); funding acquisition (equal); writing–review and editing (equal). **Dawei Zhang:** Conceptualization (equal); data curation (equal); funding acquisition (equal); project administration (equal); writing–review and editing (equal).

## Data Availability

All data generated or analyzed during this study are included in this published article.

## 1

**TABLE A1:** Experimental ranges and levels of the 11 factors tested in the Plackett–Burman design of medium optimization for the production of VB_12_ by *P*. *freudenreichii* grown at 30°C for 122 h, with pH adjustment to 7.0 every 12 h

Symbol	Factor	Unit	Range and level	
			−1	+1
A	Glucose	g/L	20	40
B	Corn steep liquor	g/L	10	30
C	Glycerol	g/L	10	20
D	–	–	–	–
E	Yeast extract	g/L	3	10
F	1%CoCl_2_·6H_2_O	μl/L	100	500
G	KH_2_PO_4_	g/L	3	5
H	–	–	–	–
I	(NH_4_)_2_SO_4_	g/L	2	4
J	Glycine	g/L	0.1	2
K	–	–	–	–

**TABLE A2:** Results of ANOVA for the Plackett–Burman design to determine which nutrient factors significantly influence VB_12_ production by *P*. *freudenreichii* grown at 30°C for 122 h, with pH adjustment to 7.0 every 12 h

Source	Coefficient in the regression equation	SS	*df*	MS	*F*‐value	*p*‐value
Model		20.94	8.00	2.62	46.79	0.0046
Intercept	4.91	–	–	–	–	–
Glucose*	0.69	5.65	1	5.65	100.92	0.0021
Corn steep liquor	0.16	0.30	1	0.30	5.36	0.1036
Glycerol	0.09	0.10	1	0.10	1.71	0.2818
Yeast extract*	1.00	11.89	1	11.89	212.58	0.0007
1%CoCl_2_·6H_2_O	0.10	0.13	1	0.13	2.31	0.2256
KH_2_PO_4_ *	−0.40	1.88	1	1.88	33.60	0.0102
(NH_4_)_2_SO_4_	0.06	0.04	1	0.04	0.70	0.4650
Glycine*	0.28	0.96	1	0.96	17.11	0.0256
Residual	–	0.17	3.00	0.06	–	–

Note

*R* ^2^ = 0.9920, Adj‐*R* ^2^ = 0.9708.

Abbreviations: *df*, degree of freedom; MS, mean square; SS, sum of squares.

* Significant medium components.

**TABLE A3:** Experimental design matrix and experimental results of the Box–Behnken design in medium optimization for the production of VB_12_ by *P*. *freudenreichii* grown at 30°C for 122 h, with pH adjustment to 7.0 every 12 h

Run	Design matrix				VB_12_ (mg/L)
	Glucose	Yeast extract	KH_2_PO_4_	Glycine	
1	−1	−1	0	0	5.011
2	1	−1	0	0	4.618
3	−1	1	0	0	5.793
4	1	1	0	0	5.431
5	0	0	−1	−1	6.806
6	0	0	1	−1	7.301
7	0	0	−1	1	7.297
8	0	0	1	1	6.849
9	−1	0	0	−1	6.110
10	1	0	0	−1	5.460
11	−1	0	0	1	6.000
12	1	0	0	1	5.909
13	0	−1	−1	0	6.193
14	0	1	−1	0	7.203
15	0	−1	1	0	5.564
16	0	1	1	0	6.790
17	−1	0	−1	0	5.810
18	1	0	−1	0	5.411
19	−1	0	1	0	5.303
20	1	0	1	0	5.109
21	0	−1	0	−1	6.402
22	0	1	0	−1	6.506
23	0	−1	0	1	6.216
24	0	1	0	1	7.598
25	0	0	0	0	8.020
26	0	0	0	0	8.212
27	0	0	0	0	8.207
28	0	0	0	0	8.308
29	0	0	0	0	8.241

Note

The coded values correspond to: for glucose (A): −1 (40 g/L), 0 (55 g/L), 1 (70 g/L); for yeast extract (B): −1 (5 g/L), 0 (15 g/L), 1 (25 g/L); for KH_2_PO_4_ (C): −1 (1 g/L), 0 (3 g/L), 1 (5 g/L); for glycine (D): −1 (1 g/L), 0 (3 g/L), 1 (5 g/L).

**TABLE A4:** Analysis of variance (ANOVA) for response surface quadratic model in medium optimization for the production of VB_12_ by *P*. *freudenreichii* grown at 30°C for 122 h, with pH adjustment to 7.0 every 12 h

Source	Coefficients of regression equation	SS	*df*	MS	*F*‐value	*p*‐value
Model	8.20	32.58	14	2.33	72.56	<0.0001
A	−0.17	0.36	1	0.36	11.34	0.0046
B	0.44	2.36	1	2.36	73.43	<0.0001
C	−0.15	0.27	1	0.27	8.46	0.0114
D	0.11	0.14	1	0.14	4.29	0.0574
AB	0.01	0.00	1	0.00	0.01	0.9334
AC	0.05	0.01	1	0.01	0.33	0.5772
AD	0.14	0.08	1	0.08	2.43	0.1412
BC	0.05	0.01	1	0.01	0.37	0.5544
BD	0.32	0.41	1	0.41	12.73	0.0031
CD	−0.24	0.22	1	0.22	6.93	0.0197
A^2^	−1.97	25.05	1	25.05	781.04	<0.0001
B^2^	−1.05	7.09	1	7.09	220.89	<0.0001
C^2^	−0.76	3.71	1	3.71	115.70	<0.0001
D^2^	−0.40	1.06	1	1.06	33.02	<0.0001
Residual		0.45	14	0.03		
Lack of Fit		0.40	10	0.04	3.52	0.1183

Note

Glucose (A), Yeast extract (B), KH2PO4 (C), Glycine (D).

*R* ^2^ = 0.9275, Adj‐*R* ^2^ = 0.9728.

Abbreviations: *df*, degree of freedom; MS, mean square; SS, sum of squares.

**TABLE A5:** Analysis of variance (ANOVA) of the reduced response surface quadratic model in medium optimization for the production of VB_12_ by *P*. *freudenreichii* grown at 30°C for 122 h, with pH adjustment to 7.0 every 12 h

Source	Coefficient in the regression equation	SS	*df*	MS	*F*‐value	*p*‐value
Model	8.2	32.48	10	3.25	106.40	<0.0001
A	−0.17	0.36	1	0.36	11.91	0.0028
B	0.44	2.36	1	2.36	77.15	<0.0001
C	−0.15	0.27	1	0.27	8.89	0.0080
D	0.11	0.14	1	0.14	4.50	0.0480
BD	0.32	0.41	1	0.41	13.38	0.0018
CD	−0.24	0.22	1	0.22	7.28	0.0147
A2	−1.97	25.05	1	25.05	820.62	<0.001
B2	−1.05	7.09	1	7.09	232.08	<0.001
C2	−0.76	3.71	1	3.71	121.56	<0.001
D2	−0.4	1.06	1	1.06	34.70	<0.001
Residual		0.55	18	0.031		
Lack of Fit		0.50	14	0.036	3.14	0.1391

Note

Glucose (A), Yeast extract (B), KH2PO4 (C), Glycine (D).

*R* ^2^ = 0.9834, Adj‐*R* ^2^ = 0.9741.

Abbreviations: SS, sum of squares; *df*, degree of freedom; MS, mean square.

**TABLE A6:** Summary of metabolites detected in metabolomics of *P*. *freudenreichii* grown with the original and optimized media at 30°C for 122 h, with pH adjustment to 7.0 every 12 h

KEGG Number	Full Name	KEGG Number	Full Name
C00002	ATP	C00148	l‐Proline
C00003	NAD+	C00149	Malate
C00004	NADH	C00152	Asparagine
C00005	NADPH	C00158	Citrate
C00006	NADP+	C00163	Propanoate
C00008	ADP	C00183	l‐Valine
C00016	FAD	C00188	l‐Threonine
C00020	AMP	C00198	Gluconolactone
C00022	Pyruvic acid	C00199	d‐Ribulose 5‐phosphate
C00024	Acetyl‐CoA	C00204	2‐Keto‐3‐deoxy‐d‐gluconate
C00025	l‐Glutamate	C00236	d‐Glycerate 1,3‐diphosphate
C00026	2‐Oxoglutarate	C00257	d‐Gluconic acid
C00031	Glucose	C00258	Glycerate
C00035	GDP	C00279	d‐Erythrose 4‐phosphate
C00037	Glycine	C00345	6‐Phosphogluconic acid
C00042	Succinate	C00407	l‐Isoleucine
C00044	GTP	C00417	cis‐Aconitate
C00047	l‐Lysine	C00577	d‐Glyceraldehyde
C00049	l‐Aspartate	C00631	2‐Phospho‐d‐glycerate
C00062	l‐Arginine	C00668	Glucose 6‐phosphate
C00064	l‐Glutamine	C01151	d‐Ribose 1,5‐bisphosphate
C00065	Serine	C01172	beta‐d‐Glucose 6‐phosphate
C00073	Methionine	C01213	Methylmalonyl‐CoA
C00074	Phosphoenolpyruvate	C01218	2‐Dehydro‐d‐gluconate 6‐phosphate
C00078	Tryptophan	C01236	d‐Glucono‐1,5‐lactone 6‐phosphate
C00082	Tyrosine	C01352	FADH2
C00091	Succinyl‐CoA	C02876	Propanoyl phosphate
C00097	l‐Cysteine	C03752	d‐Glucosaminate
C00100	Propanoyl‐CoA	C04442	2‐Dehydro‐3‐deoxy‐6‐phospho‐d‐gluconate
C00117	d‐Ribose 5‐phosphate	C05345	beta‐d‐Fructose 6‐phosphate
C00118	Glyceraldehyde 3‐phosphate	C05378	beta‐d‐Fructose 1,6‐bisphosphate
C00119	5‐Phosphoribosyl diphosphate	C05382	d‐Sedoheptulose 7‐phosphate
C00122	Fumarate	C06473	2‐Dehydro‐d‐gluconate
C00135	l‐Histidine	C20589	d‐Glucosaminate‐6‐phosphate
C00144	GMP		

**TABLE A7:** List of differential metabolites at the logarithmic phase, stationary phase, and VB_12_ production phase. log2 (FC) means the log base twofold change values. Differential metabolites were identified using the following criteria: log2 (FC) >1 or <−1, and −log(P) > 1.301 (*p*‐value < 0.05).

Logarithmic phase			Stationary phase			VB_12_ production phase		
Metabolites	log_2_ (FC)	‐lg(P)	Metabolites	log_2_ (FC)	‐lg(P)	Metabolites	log_2_ (FC)	‐lg(P)
2‐Dehydro‐3‐deoxy‐d‐gluconate	2.05367	4.49607	2‐Dehydro−3‐deoxy‐d‐gluconate	−2.51145	7.76662	6‐Phosphogluconic acid	−3.26569	3.17945
2‐Keto‐gluconic acid	1.24351	2.79056	Acetyl‐CoA	3.18808	2.65468	6‐Phosphogluconic acid	−3.26569	3.17945
Acetyl‐CoA	4.35585	2.48321	Aconitic acid	−1.0933	2.46648	Adenosine monophosphate	1.89134	2.96532
Adenosine monophosphate	2.37507	4.74851	Adenosine monophosphate	1.69077	1.84774	Aspartic acid	1.61263	3.45256
Arginine	3.4152	2.49041	Adenosine triphosphate	1.23665	1.63361	Aconitic acid	−2.20843	4.49006
Aspartic acid	4.06933	4.1856	Aspartic acid	1.89534	2.28934	FAD	1.10727	2.00782
cis‐Aconitic acid	2.47559	2.69331	Citric acid	−2.57374	3.50397	FADH	1.36122	2.59461
Citric acid	2.10141	3.14467	FAD	1.10237	2.51284	Fructose 1,6‐bisphosphate	−1.45398	2.85088
Cysteine	−3.46801	2.38333	FADH	1.14113	2.36028	Fumaric acid	−2.1796	2.48359
FAD	1.3632	2.03838	Fructose 1,6‐bisphosphate	−1.56051	3.566	Guanosine monophosphate	−4.57931	2.38568
FADH	2.14206	1.81123	Gluconic acid	−2.54297	2.267	Malic acid	−2.20661	2.4959
Fructose 6‐phosphate	2.40673	4.0459	Gluconolactone	−2.51145	7.76662	Methionine	−1.3884	3.28576
Fumaric acid	2.54599	3.54268	Glucose	−2.26019	3.84174	Methylmalonyl‐CoA	1.32167	3.4627
Gluconic acid	2.2496	4.45531	Glutamic acid	1.07994	2.85799	NADP	1.28716	3.32016
Gluconolactone	2.05367	4.49607	Glyceraldehyde	−2.7688	3.51544	Oxoglutaric acid	−4.48178	3.18444
Glucose	2.61453	3.92834	Guanosine monophosphate	−4.51203	3.06703	Phosphoribosyl pyrophosphate	−2.74597	2.4644
Glucose 6‐phosphate	2.40673	4.0459	Methylmalonyl‐CoA	1.87291	4.16991	Ribose 1,5‐bisphosphate	−3.25943	3.71755
Glutamic acid	5.45949	4.33264	Oxoglutaric acid	−3.20191	3.13281	Ribose 5‐phosphate	−3.1367	4.83038
Glyceraldehyde	2.21778	3.81563	Phosphoribosyl pyrophosphate	−2.37776	2.4204	Ribulose 5‐phosphate	−3.1367	4.83038
Glyceraldehyde 3‐phosphate	2.21411	2.80225	Propionyl‐CoA	4.15035	2.97379	Sedoheptulose 7‐phosphate	−1.68031	4.5191
Glyceric acid	1.82435	1.53855	Pyruvic acid	−3.52997	6.48381	Succinyl‐CoA	1.32167	3.4627
Glyceric acid 1,3‐biphosphate	2.24973	1.53222	Ribose 1,5‐bisphosphate	−2.79329	2.99855	Tyrosine	1.14909	2.01647
Glycine	2.77207	3.11141	Ribose 5‐phosphate	−1.59255	2.6474	Valine	1.42203	1.81475
Guanosine diphosphate	1.16527	1.88233	Ribulose 5‐phosphate	−1.59255	2.6474			
Histidine	−3.46801	2.38333	Succinyl‐CoA	1.87291	4.16991			
Isoleucine	3.23544	2.85877	Valine	1.27713	3.34286			
Lysine	3.24613	1.92555						
Malic acid	2.48498	3.51363						
Methionine	3.7114	6.15925						
Methylmalonyl‐CoA	3.26596	2.73429						
Oxoglutaric acid	3.27512	4.91939						
Phosphoribosyl pyrophosphate	−1.3172	2.85536						
Proline	3.2362	10.1264						
Propanoyl phosphate	5.17919	1.64887						
Propionic acid	2.29647	2.02239						
Propionyl‐CoA	6.45464	2.72025						
Pyruvic acid	1.98894	3.41543						
Sedoheptulose 7‐phosphate	1.03178	1.73171						
Serine	4.3842	2.44302						
Succinic acid	3.38252	5.73543						
SuccinyCoA	3.26596	2.73429						
Threonine	1.37103	1.32208						
Tryptophan	5.26622	5.09113						
Tyrosine	4.1815	3.85977						
Valine	3.77485	4.81414						

**TABLE A8:** l‐Metaboanalyst pathway analysis of upregulated metabolites at the logarithmic phase

Pathway name	*p*‐value	FDR	Impact
Propanoate metabolism	3.26E−06	7.10E−05	0.78378
Glycine, serine, and threonine metabolism	1.02E−04	0.001726	0.49492
Alanine, aspartate, and glutamate metabolism	3.44E−04	0.003327	0.47873
Butanoate metabolism	3.44E−04	0.003327	0.45098
Citrate cycle (TCA cycle)	1.69E−07	7.86E−06	0.39852
Glycolysis or Gluconeogenesis	0.0035169	0.021898	0.34039
Valine, leucine and isoleucine degradation	1.19E−04	0.001726	0.23529
Arginine and proline metabolism	0.0032127	0.021898	0.20052
Glyoxylate and dicarboxylate metabolism	4.74E−04	0.004124	0.1884
Pentose phosphate pathway	1.78E−06	5.17E−05	0.16295
Aminoacyl‐tRNA biosynthesis	1.81E−07	7.86E−06	0.13043
Valine, leucine, and isoleucine biosynthesis	0.013998	0.076117	0.0356

Note

FDR means false discovery rate.

**TABLE A9:** Metaboanalyst pathway analysis of upregulated metabolites at the stationary phase

Pathway name	*p*‐value	FDR	Impact
Valine, leucine, and isoleucine degradation	1.63E−06	1.42E−04	0.23529
Propanoate metabolism	3.38E−05	0.0014695	0.59459
Nitrogen metabolism	7.17E−04	0.020786	0

Note

FDR means false discovery rate.

**TABLE A10:** Metaboanalyst pathway analysis of downregulated metabolites at the stationary phase

Pathway name	*p*‐value	FDR	Impact
Pentose phosphate pathway	9.3292E−12	8.1164E−10	0.30652
Citrate cycle (TCA cycle)	9.8937E−4	0.043038	0.16139

Note

FDR means false discovery rate.

**TABLE A11:** Metaboanalyst pathway analysis of downregulated metabolites at the VB_12_ production phase

Pathway name	*p*‐value	FDR	Impact
Pentose phosphate pathway	6.6786E−8	5.8104E−6	0.41544
Citrate cycle (TCA cycle)	9.8937E−4	0.043038	0.10827

Note

FDR means false discovery rate.
